# Developmental transition of *Plasmodium* sporozoites into liver-stage forms is regulated by the RNA binding protein Pumilio 2

**DOI:** 10.1186/1475-2875-9-S2-P13

**Published:** 2010-10-20

**Authors:** Carina SS Gomes-Santos, J Anneke M Braks, Miguel Prudêncio, Céline K Carret, Ana Rita Gomes, Arnab Pain, Theresa Feltwell, Shahid M Khan, Andrew P Waters, Chris J Janse, Gunnar R Mair, Maria M Mota

**Affiliations:** 1 Malaria Unit, Instituto de Medicina Molecular, Faculdade de Medicina Universidade de Lisboa, 1649-028 Lisboa, Portugal; 2 PhD Programme in Experimental Biology and Biomedicine, Center for Neuroscience and Cell Biology, University of Coimbra, 3004-517 Coimbra, Portugal; 3 Laboratory for Parasitology, Leiden University Medical Centre, 2333 ZA Leiden, The Netherlands; 4 Unidade de Parasitologia Molecular, Instituto de Medicina Molecular, 1649-028 Lisboa, Portugal; 5 Pathogen Genetics Group, Wellcome Trust Sanger Institute, Cambridge, UK; 6 Division of Infection and Immunity, Institute of Biomedical Life Sciences & Wellcome Centre for Molecular Parasitology, Glasgow Biomedical Research Centre, University of Glasgow, Glasgow G12 8TA, UK

## 

Developmental and cell fate decisions in many organisms are frequently made on a post-transcriptional level that involves RNA binding proteins and mechanisms of translational repression and regulation of translation of key mRNAs. In the unicellular eukaryote Plasmodium, one of the most dramatic life-cycle dependent changes in cell morphology and function occurs during transmission from the mosquito to the human host. In the mosquito salivary glands Plasmodium sporozoites remain slender, motile and infectious for several days/weeks. At this stage, Plasmodium parasites are on stand-by, awaiting injection onto the mammalian host. Immedietaly fter transmission and liver cell invasion, a remarkable differentiation process is initiated. During this process, each parasite rapidly transforms into a round, stationary exo-erythrocytic form (EEF) that expands into thousands of infectious merozoites to be released into the blood stream. Here we reveal a Plasmodium homolog of the RNA binding protein Pumilio as a key regulator of the sporozoite to EEF transition. In the absence of Pumilio-2 (Puf2), Plasmodium berghei sporozoites initiate early stage EEF development inside mosquito salivary glands, in the absence of the appropriate environmental cues. Mutant salivary gland sporozoites present reduced motility but also significantly reduced ability to invade and infect their mammalian host cells, both in vitro and in vivo. Global expression profiling confirmed that transgenic parasites exhibit genome-wide transcriptional adaptations that are typical for Plasmodium intra-hepatic development. Our data, demonstrates that Puf2 is a key player in the developmental control involved in the transition between sporozoite and EEF, implying that transformation of salivary gland-resident sporozoites into early liver stage parasites is in fact prevented by a post-translational mechanism.

